# Improving attribution of extreme heat deaths through interagency cooperation

**DOI:** 10.17269/s41997-022-00672-2

**Published:** 2022-08-11

**Authors:** Sarah B. Henderson, Félix Lamothe, Jiayun Yao, Celine Plante, Shawn Donaldson, Rebecca Stranberg, David Kaiser, Tom Kosatsky

**Affiliations:** 1 Environmental Health Services, BC Centre for Disease Control, Vancouver, BC Canada; 2 Environnements urbains et santé des populations, Direction régionale de santé publique de Montréal, Montréal, QC Canada; 3 Healthy Environments and Consumer Safety Branch, Health Canada, Ottawa, ON Canada

**Keywords:** Extreme heat event, Mortality attribution, Public health, Climate change, Épisode de chaleur extrême, attribution de la mortalité, santé publique, changement climatique

## Abstract

Attributing individual deaths to extreme heat events (EHE) in Canada and elsewhere is important for understanding the risk factors, protective interventions, and burden of mortality associated with climate change. However, there is currently no single mechanism for identifying individual deaths due to EHE and different agencies have taken different approaches, including (1) vital statistics coding based on medical certificates of death, (2) probabilistic methods, and (3) enhanced surveillance. The 2018 EHE in Montréal provides an excellent case study to compare EHE deaths identified by these different approaches. There were 353 deaths recorded in the vital statistics data over an 8-day period, of which 102 were potentially attributed to the EHE by at least one approach and 251 were not attributed by any approach. Only nine of the 102 deaths were attributed to the EHE by all three approaches, 23 were attributed by two approaches, and 70 were attributed by only one approach. Given that there were approximately 50 excess deaths during the EHE, it remains unclear exactly which of the total 353 deaths should be attributed to the extreme temperatures. These results highlight the need for a more systematic and cooperative approach to EHE mortality in Canada, which will continue to increase as the climate changes.

## Résumé

L’attribution des décès individuels aux épisodes de chaleur accablante (ECA) au Canada et ailleurs est importante pour comprendre les facteurs de risque, les interventions de protection et le fardeau de la mortalité associés aux changements climatiques. Cependant, il n’existe actuellement aucun mécanisme unique pour identifier les décès individuels dus à l’ECA et différentes agences ont adopté différentes approches, notamment (1) le codage des statistiques de l’état civil basé sur les certificats médicaux de décès, (2) des méthodes probabilistes et (3) une surveillance renforcée. L’ECA 2018 à Montréal fournit une excellente étude de cas pour comparer les décès ECA identifiés par ces différentes approches. Il y a eu 353 décès enregistrés dans les données des statistiques de l’état civil sur une période de 8 jours, dont 102 ont été potentiellement attribués à l’ECA par au moins une approche et 251 n’ont été attribués par aucune approche. Seuls neuf des 102 décès ont été attribués à l’ECA par les trois approches, 23 ont été attribués par deux approches et 70 ont été attribués par une seule approche. Étant donné qu’il y a eu environ 50 décès supplémentaires pendant l’ECA, on ne sait pas exactement lequel des 353 décès au total doit être attribué aux températures extrêmes. Ces résultats soulignent la nécessité d’une approche plus systématique et coopérative de la mortalité ECA au Canada, qui continuera d’augmenter à mesure que le climat change.

## Introduction

Extreme heat events (EHE) are often referenced as a *silent killer* because they have a marked impact on population mortality, but the deaths go relatively unnoticed compared with those caused by other exposures of public health interest. For example, the 2009 EHE in greater Vancouver was associated with approximately 110 excess deaths over a 7-day period (Kosatsky et al., 2012), whereas only 62 deaths in the province of British Columbia (BC) were attributed to the following H1N1 pandemic. Furthermore, those deaths were all directly attributed to H1N1 in the individual vital statistics records using the International Classification of Diseases, 10^th^ Revision (ICD-10) code J09.X2. In comparison, only one of the 411 deaths during the EHE was directly attributed the ICD-10 code X30, indicating exposure to excessive natural heat. As such, we estimate that approximately 110 people died because of the EHE, but we cannot identify the specific individuals. National vital statistics data indicate that an average of only 16 deaths per year are coded as X30 (Statistics Canada, 2021).

Attributing individual deaths to EHE is important for three reasons. First, it is difficult to narrow down the specific risk factors for hot weather mortality when retrospective analyses also include many expected deaths over the EHE period. We generally know that social isolation, economic status, lack of greenspace, age, comorbidities, and some drugs are associated with increased risk, but more refined data would lead to more specific conclusions (Cheng et al., 2021). Second, EHE will become more frequent and intense across Canada as the global climate changes. Building population resiliency to these events requires targeted public health interventions based on known risk factors (Kafeety et al., 2020). Third, there is a growing need to quantify the health effects of EHE in the same way as other meteorological events, such as tropical storms, to ensure that analogous emergency responses are developed and resourced (Henderson et al., 2021; Mitchell et al., 2016). With these objectives in mind, different agencies have taken different approaches to hot weather attribution of individual deaths in Canada. Here we present a comparative case study of three independent approaches applied to the same EHE.

## Approach 1: Vital statistics coding

Vital statistics agencies across Canada receive certificates of death from clinicians and coroners, and systematically translate these documents into data that are used for provincial and national reporting on mortality. The underlying causes of death recorded on the certificates must be interpreted and coded according to standard protocols (US National Vital Statistics System, 2020), which can require an iterative process of further discussions with the certifier. Technically, the ICD-10 code X30 should only be recorded as the underlying cause when high temperatures are solely responsible for an accidental and preventable death. In other words, the death would not have occurred in the absence of high temperatures. However, many deaths during EHE occur among highly susceptible individuals who are at higher risk due to age and comorbidities, and this makes it challenging to conclude that heat was the causal factor. While the code T67 may also be used to record heat exposure as a contributing cause of death, these secondary fields are not included in national vital statistics reporting on mortality.

## Approach 2: Probabilistic methods

After the 2009 EHE in greater Vancouver, the BC Centre for Disease Control (BCCDC) developed a statistical approach to separate excess deaths from expected deaths, which is described in detail elsewhere (Henderson et al., 2016). In brief, the number of expected deaths (N) during the EHE is calculated and N vital statistics records are randomly and repeatedly resampled from the records of all deaths observed during the EHE. The sampled EHE deaths are then matched to comparators randomly sampled from records of deaths that occurred during typical summer weather.

Conditional logistic regression is used to evaluate differences between the EHE deaths and the comparison group for five risk factors known to be associated with hot weather mortality: age, setting of death (e.g., hospital), population density, neighbourhood deprivation, and surrounding residential greenness (Kosatsky et al., 2012). Deaths during the EHE that consistently appear in models that are significantly different from the typical weather deaths are identified and ranked, the lowest N deaths are taken as those most probably expected, and the rest are attributable to the extreme temperatures. While this approach can provide valuable insight into deaths due to EHE, the results are only available to those who apply the method, and they are not reflected in provincial or national vital statistics data.

## Approach 3: Enhanced surveillance

After a deadly 2010 EHE in Montréal, the Direction régionale de santé publique (DRSP) developed an enhanced surveillance protocol for identifying deaths attributable to extreme temperatures (Price et al., 2018). In brief, the system is activated whenever the city issues an EHE alert. Once the alert has ended, the DRSP conducts a review of all deaths during the EHE. Using all available records, details regarding the location of death, ambient temperature, air conditioning, and known comorbidities of the decedent are entered into the enhanced surveillance form. A decision tree, updated in 2018, is used to classify each death as being *confirmed*, *probable*, *unlikely*, or *indeterminate* with respect to EHE attribution.

Only deaths with a recorded body temperature > 40 °C are classified as *confirmed*. *Probable* is used when the temperature at the time and place of death was elevated and the cause was a condition associated with heat-related mortality, such as myocardial infarction or neurological disorders. *Unlikely* is used for deaths due to accidents, trauma, suicide, homicide, advanced cancer, surgical complications, or when there was evidence of air conditioning at the place of death. *Indeterminate* is used for deaths with no information about ambient temperature or air conditioning and due to a cause not included in the *confirmed* or *unlikely* categories. Once again, these results are only available to DRSP, and they are not reflected in any vital statistics data.

## Cross-comparison of approaches

**Figure 1:**
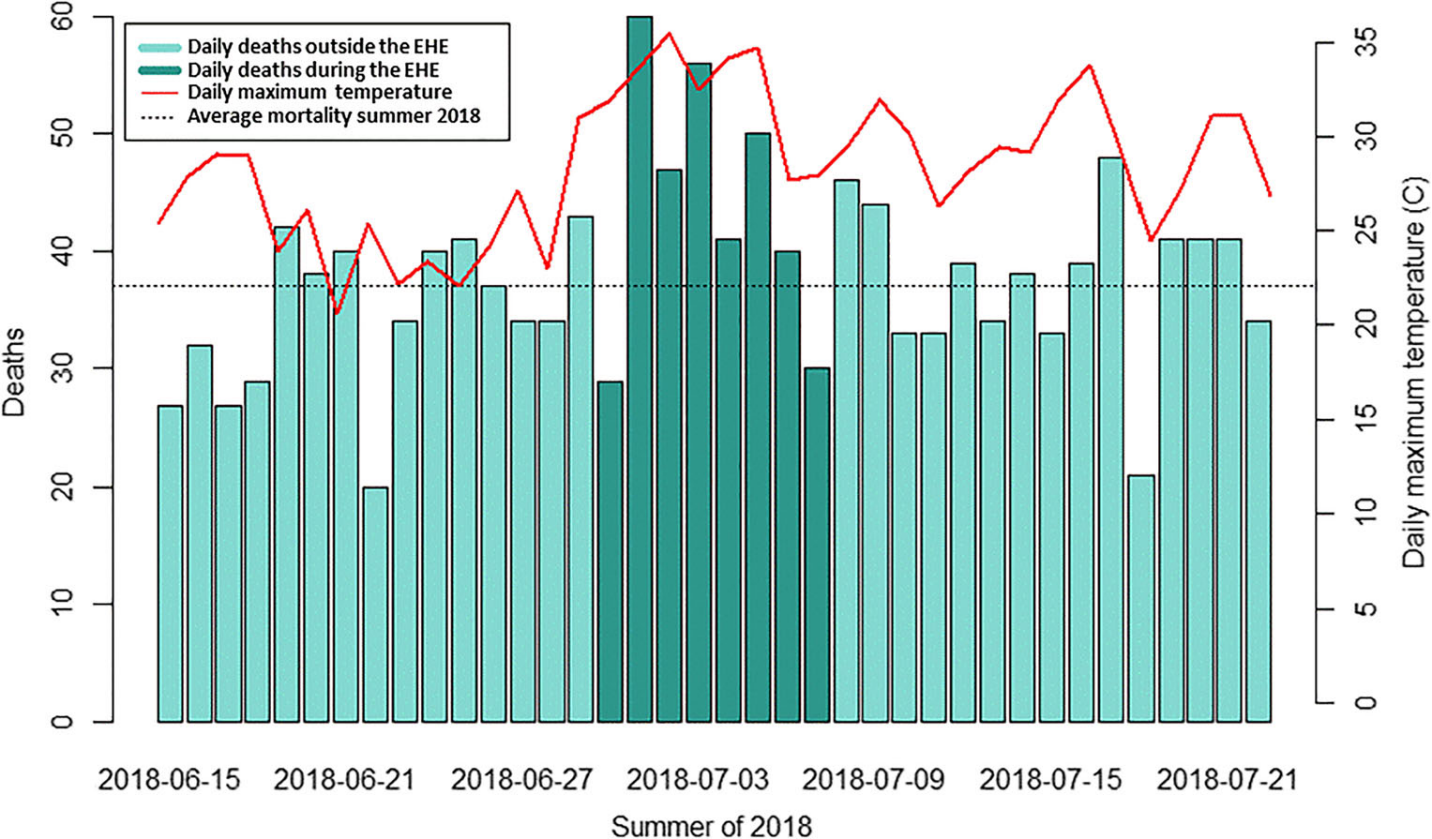
Daily deaths and temperatures during the Montréal extreme heat event (EHE) in the summer of 2018. The maximum temperatures measured at Pierre Elliott Trudeau Airport are shown on the right-hand axis, and the 353 deaths that occurred during the 8-day EHE are shown as darker bars

**Figure 2:**
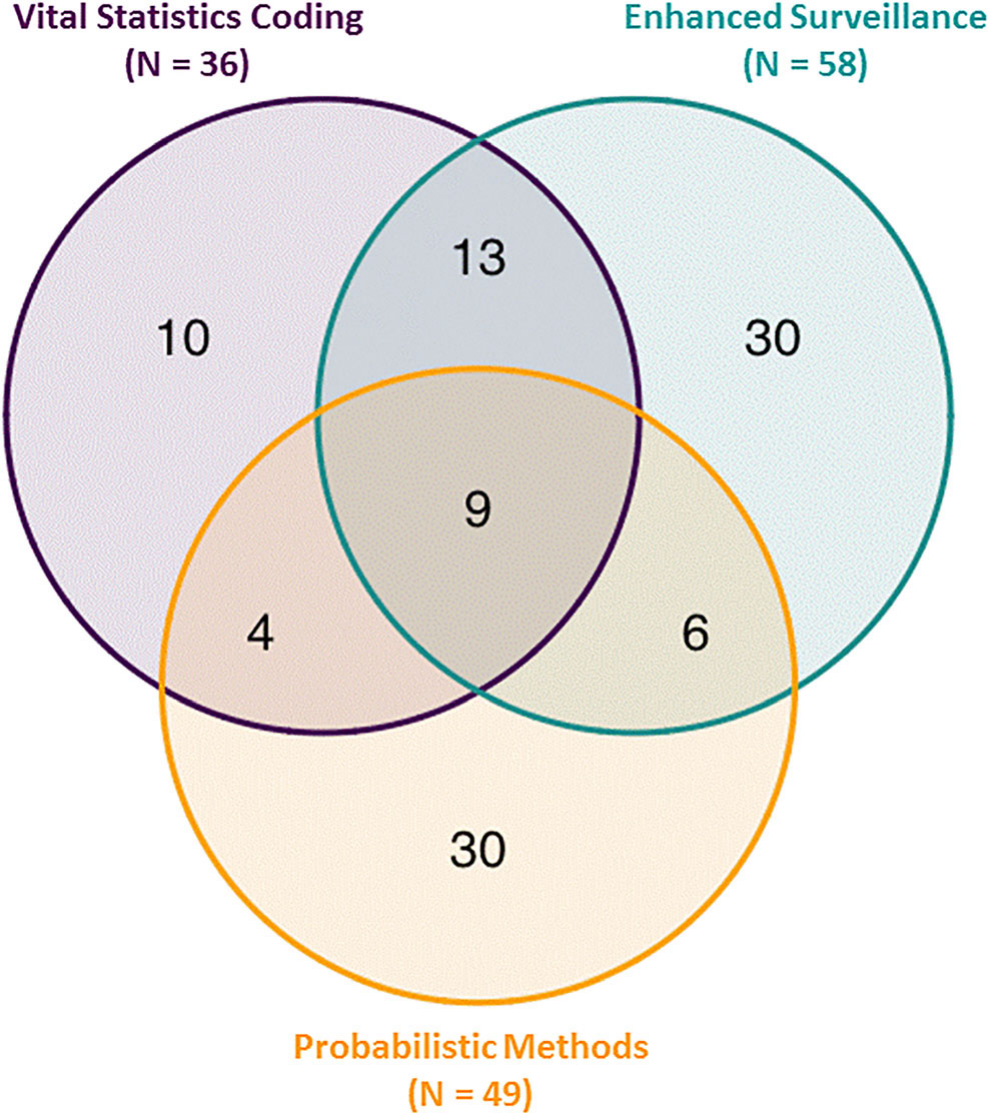
Venn diagram showing the overlap in deaths attributed to the 2018 extreme heat event (EHE) in Montréal by three different approaches. There were 102 deaths attributed to the EHE by at least one approach, and 251 deaths not attributed to the EHE by any approach

The city of Montréal was under an EHE alert from 30 June through 07 July 2018, which provides an excellent opportunity to compare these three approaches to mortality attribution. There were 353 deaths recorded in the vital statistics database during the 8-day period (Figure 1). Of these, 36 had the ICD-10 code X30 listed as the underlying cause of death (Approach 1) and none had the code T67 indicated. There were an estimated 49 excess deaths during the EHE, and the probabilistic algorithm was applied to separate them from the 304 expected deaths (Approach 2). The enhanced surveillance form was completed by DRSP for 328 deaths, of which 58 were classified as having *confirmed* or *probable* attribution to the EHE and could be linked to the vital statistics records (Approach 3). Together, these methods identified a total of 102 individual deaths that may have been attributable to the EHE. However, only 32 of the deaths were identified by more than one approach while 70 were identified by a single approach (Figure 2).

We found a large shift towards deaths at home when the 102 deaths potentially attributed to the EHE (55.9% at home) were compared with the unattributed 251 deaths (10.8% at home), which is a hallmark of EHE mortality (Hajat et al., 2010). When the 30 deaths only attributed by the probabilistic approach were compared with the 30 deaths only attributed by enhanced surveillance, there were some important differences with respect to underlying cause of death. The probabilistic approach identified 17 deaths due to neoplasms and accidental causes, which were largely excluded by the DRSP decision tree. On the other hand, the enhanced surveillance identified 22 deaths due to diseases of the circulatory system whereas the probabilistic approach only identified four (Table 1).

**Table 1:** Comparison of deaths only identified by (1) the probabilistic methods developed by the British Columbia Centre for Disease Control (BCCDC) and (2) the enhanced surveillance methods developed by the Direction régionale de santé publique de Montréal (DRSP)

	Probabilistic methods only	Enhanced surveillance only
**Total**	**30**	**30**
Neoplasms (C00-D48)	12	1
Mental and behavioural disorders (F00-F99)	-	2
Disease of the circulatory system (I00-I99)	4	22
Disease of the digestive system (K00-K93)	3	-
External causes of mortality (V01-Y98)	5	1
Other	4	-
Unknown at this time (R99)	2	4

## Discussion

The number of deaths attributable to any given EHE is generally calculated using statistical estimates of expected and excess mortality (Henderson et al., 2021). Under ideal circumstances, the number of excess deaths estimated for an EHE would be consistent with the number of deaths in provincial and national vital statistics records with an ICD-10 code of X30 or T67 during the same period. Furthermore, there would be perfect agreement between the EHE deaths identified by vital statistics, probabilistic methods, and enhanced surveillance when multiple approaches are available.

This case study highlights the value of having vital statistics records with the X30 code while demonstrating that different approaches to attributing individual EHE mortality produce somewhat discordant results. We found that 102 of 353 individual deaths were attributed by at least one of three approaches, and approximately 50 of these deaths were excess based on statistical estimates. There were 36 deaths with an X30 code, 49 attributed by the probabilistic method, and 58 identified through enhanced surveillance. The overlap between two or more approaches was limited to 32 deaths, and 70 were only identified by a single approach. We also found discrepancies in the types of deaths identified by the probabilistic and enhanced surveillance approaches, suggesting that both may have missed some important indicators of deaths due to EHE.

Each approach has strengths and limitations. It is critically important to have appropriate ICD-10 codes reflected in national vital statistics records so that deaths due to EHE can be identified in the same way as deaths due to most other causes in Canada. However, cause of death information is subject to significant delays in vital statistics data and cannot facilitate the near-real-time analyses possible with the probabilistic and enhanced surveillance methods described here. The ideal future state may combine these types of approaches to ensure that rapid assessments are possible, and that vital statistics records appropriately reflect individual deaths due to EHE.

The experience in BC during the 2021 heat dome provides a framework on which a more systematic future approach could be modelled. Early in the event, the BC Coroners Service (BCCS) sent a reminder to clinicians that any death with extreme heat as a possible contributor should be reported for investigation. Soon after the event, the BCCDC estimated 740 excess deaths during the EHE (Henderson et al., 2021) and conducted epidemiologic analyses on the deaths most likely due to the extreme heat (Henderson et al., 2022). Since then, BCCS, BC Vital Statistics, and BCCDC have worked together to ensure that the mortality impacts of the EHE are well understood and that most deaths will be appropriately attributed. To date, the BCCS has confirmed at least 619 heat-related deaths that will be recorded with X30 or T67 in the provincial vital statistics data (BC Coroners Service, 2022).

More generally, Environment and Climate Change Canada provides hot weather notifications across the country. Such notifications could be used to trigger regional public health reminders for clinicians and coroners to consider the role of high temperatures in any deaths they attend. If heat may have been a causal or contributing factor, noting it on the certificate of death creates the opportunity for future review and appropriate attribution. A death simply cannot be coded as X30 or T67 by vital statistics agencies unless this information is included on the certificate of death they receive. Coroners, clinicians, public health, vital statistics, and other agencies could then work together after the event to review specific cases, especially those where the role of heat is less clear. This type of systematic and cooperative approach would facilitate improved understanding of EHE and the burden of mortality, risk factors, and potentially effective interventions in Canada.

## Data Availability

Not available.

## Code availability

Upon request.
